# Occurrence of the Microcystins MC-LW and MC-LF in Dutch Surface Waters and Their Contribution to Total Microcystin Toxicity

**DOI:** 10.3390/md11072643

**Published:** 2013-07-22

**Authors:** Elisabeth J. Faassen, Miquel Lürling

**Affiliations:** 1 Aquatic Ecology & Water Quality Management Group, Wageningen University, PO Box 47, Wageningen 6700 AA, The Netherlands; E-Mail: miquel.lurling@wur.nl; 2 Department of Aquatic Ecology, Netherlands Institute of Ecology, Droevendaalsesteeg 10, Wageningen 6708 PB, The Netherlands

**Keywords:** cyanobacterial bloom, cyanotoxins, hepatotoxins, LC-MS/MS, surface scum

## Abstract

Microcystins (MCs) are the most frequently found cyanobacterial toxins in freshwater systems. Many MC variants have been identified and variants differ in their toxicity. Recent studies showed that the variants MC-LW and MC-LF might be more toxic than MC-LR, the variant that is most abundant and mostly used for risk assessments. As little is known about the presence of these two variants in The Netherlands, we determined their occurrence by analyzing 88 water samples and 10 scum samples for eight MC variants ((dm-7-)MC-RR, MC-YR, (dm-7-)MC-LR, MC-LY, MC-LW and MC-LF) by liquid chromatography with tandem mass spectrometry detection. All analyzed MC variants were detected, and MC-LW and/or MC-LF were present in 32% of the MC containing water samples. When MC-LW and MC-LF were present, they contributed to nearly 10% of the total MC concentrations, but due to their suspected high toxicity, their average contribution to the total MC toxicity was estimated to be at least 45%. Given the frequent occurrence and possible high toxicity of MC-LW and MC-LF, it seems better to base health risk assessments on the toxicity contributions of different MC variants than on MC-LR concentrations alone.

## 1. Introduction

**Figure 1:**
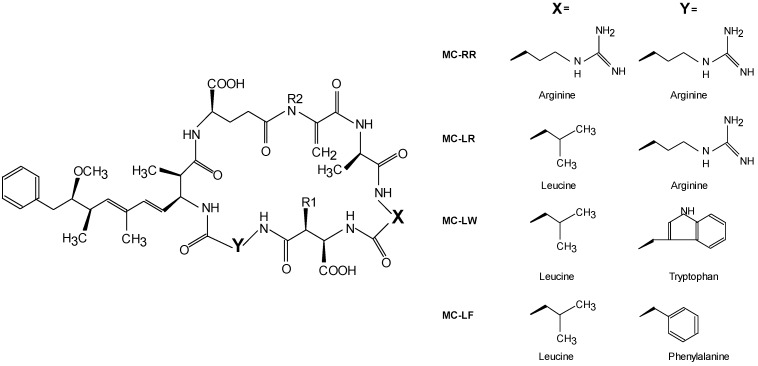
General structure of microcystins and examples of substitutions at position X and Y resulting in the variants MC-RR, MC-LR, MC-LW and MC-LF if positions R1 and R2 are methylated.

The incidence and intensity of cyanobacterial blooms are on the rise worldwide [1,2]. Cyanobacterial blooms present a serious health threat because cyanobacteria might produce potent toxins [3] of which microcystins (MCs) are most frequently encountered in freshwater blooms all around the world [4]. MCs are non-ribosomally synthesized cyclic heptapeptides with a size between 909 and 1115 Da [4,5]. Their general structure is cyclo(-d-ala-l-X-erythro-β-d-methylaspartic acid-l-Y-Adda-d-isoglutamic acid-*N*-methyldehydroalanine), where Adda is (2*S*,3*S*,8*S*,9*S*)-3-amino-9-methoxy-2,6,8-trimethyl-10-phenyldeca-4,6-dienoic acid and X and Y are variable l-amino acids [6] (Figure 1).

MCs are potent inhibitors of protein phosphatases and they primary cause liver damage, although other organs can also be affected [7]. Furthermore, MCs are tumor promoters [7,8]. The toxicity of different variants when administered by intraperitoneal injection (i.p.) to mice varies substantially. MC-LR is the most toxic variant and replacement of the hydrophobic leucine (L) in position X with a more hydrophilic amino acid (e.g., arginine, R) reduces toxicity [8]. No mouse toxicity data are available for MC-LW and MC-LF, but recent *in vitro* studies show that these variants, in which the hydrophobic amino acids tryptophan (W) or phenylalanine (F) occupy position Y, were distinctly more toxic than MC-LR. They were more cytotoxic than MC-LR to human hepatocytes, organic anion transporting polypeptide (OATP)-transfected embryonic kidney cells and Caco-2 cells [9,10] and also to primary murine whole brain cells [11]. Also when other endpoints were used, like tau phosphorylation and neurite length of murine neurons [12], proliferation and morphology of human Caco-2 cells [10] and growth inhibition in human OATP-transfected HeLa cells [13], MC-LW and MC-LF were distinctly more toxic than MC-LR. Because MC-LW and MC-LF were approximately equally strong protein phosphatase inhibitors as MC-LR [9,10,13], their enhanced toxicity can probably be attributed to the greater ability of these variants to enter cells, either because of variant dependent OATP mediated transport [14] or because of differences in the interaction with membranes [15]. MC-LW and MC-LF were also more toxic to the protozoan *Tetrahymena pyriformis* than MC-LR [16], although this effect was less pronounced than in the *in vitro* studies (Table 1).

**Table 1:** Relative toxicity of seven microcystin variants to MC-LR based on different endpoints, higher numbers indicate higher toxicity. i.p.: Intraperitoneal injection; dm-7: demethylated at position R2; n.d.: Not determined.

MC variant	LD_50_ mouse (i.p.) [8]	LC_50_ protozoan [16]	EC_50_ *in vitro* ^1^ [9]	Factor used in this study
dm-7-MC-RR	0.28	n.d.	n.d.	0.28
MC-RR	0.06–0.10	n.d.	0.02–0.20	0.08
MC-YR	0.25–0.33	n.d.	n.d.	0.29
dm-7-MC-LR	0.20	n.d.	n.d.	0.20
MC-LY	0.56	1.1–1.4	n.d.	0.56
MC-LW	n.d.	1.9–2.9	7–64	7
MC-LF	n.d.	2.0–3.0	7–69	7

^1^ Based on the cytotoxicity to human primary hepatocytes and OATP-transfected human embryonic kidney cells.

Few reports exist on the occurrence of MC-LW and MC-LF in environmental samples. MC-LR is one of the most frequently observed MCs in the environment along with the variants MC-RR and MC-YR [4]. However, when looked for, MC-LW and MC-LF have also been detected [17,18,19,20,21,22,23,24,25,26], albeit not in all cases (e.g., [27,28,29]). Despite the potential higher toxicity of MC-LW and MC-LF, risk assessments are often based on MC-LR or MC-LR equivalents, and only sometimes on total MC or MC-LR toxicity equivalents [30].

**Figure 2:**
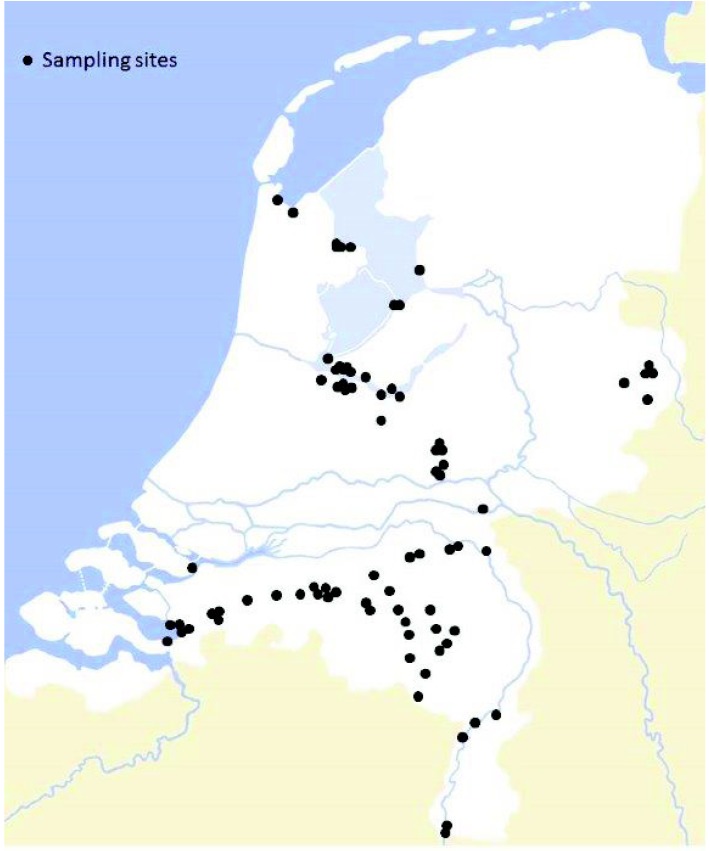
Sampling sites in the Netherlands.

In The Netherlands, MCs are the most abundant cyanotoxins, but little information exists on the occurrence of different variants, as most MC analyses have been performed by ELISA. Occasionally, some environmental samples have been analyzed by high-performance liquid chromatography with photodiode array detection but only for the variants MC-LR, MC-RR and MC-YR [31]. However, recently we showed that considerable amounts of the variants MC-LW and MC-LF were present in cyanobacterial material from a case of dog fatalities in The Netherlands [25]. The aim of the current research was therefore to determine the occurrence of MC-LW and MC-LF in Dutch surface waters and to estimate their contribution to the total MC toxicity. For this, 88 water samples and 10 scum samples from 86 sites in The Netherlands (Figure 2) were analyzed for eight MC-variants and nodularin by liquid chromatography with tandem mass spectrometry detection (LC-MS/MS).

## 2. Results and Discussion

Microcystins were detected in 77 out of 88 water samples analyzed. In four samples MCs were present only at trace levels, but in 73 samples in sufficient amounts to allow quantification (Table 2). MC-LR was the most frequently encountered variant being present in 85% of the samples, followed by MC-YR (82%), dm-7-MC-LR (81%), MC-RR (78%) and dm-7-MC-RR (66%). MC-LY (28%), MC-LW (23%) and MC-LF (26%) were less frequently detected and mostly occurred together (Supplementary Table S1 and Table 2). *Microcystis aeruginosa* or *M. flos-aquae* were dominantly present in all but one location where MC-LW and MC-LF were detected. The other location where MC-LF was present was dominated by *Woronichinia naegeliana* and *Anabaena flos-aquae* (Supplementary Table S1). MC-LR, dm-7-MC-LR, MC-YR and MC-RR were also most frequently present in the cyanobacterial scums as they were detected in each sample. dm-7-MC-RR was detected in eight of the 10 samples and MC-LY, MC-LW and MC-LF were again less frequently detected, they were encountered in four of the 10 samples (Table 3). Also, in the scums, MC-LY, MC-LW and MC-LF were mostly detected together and they were detected at locations where either *M. aeruginosa*, *W. naegeliana* or *A. flos-aquae* was present (Supplementary Table S2).

**Table 2:** Microcystin (MC) concentrations (μg/L) detected in 88 water samples, summarized from Supplementary Table S1. Nodularin was not detected.

	dm-7-RR	RR	YR	dm-7-LR	LR	LY	LW	LF	Total MC
average	0.45	5.0	2.4	4.0	35	5.9	14	4.2	49
median	0.20	0.81	0.23	0.12	1.3	0.35	0.61	0.42	2.5
maximum	3.6	66	41	220	2100	110	260	33	2800
minimum	0.03	0.02	0.01	0.003	0.01	0.001	0.05	0.05	0.02
*n* quantified	38	67	67	62	73	25	20	17	73
*n* not quantified	20	2	5	9	2	0	0	6	4
*n* not detected	30	19	16	17	13	63	68	65	11

**Table 3:** Microcystin (MC) concentrations (μg/L) detected in 10 scum samples, summarized from Supplementary Table S2. Nodularin was not detected.

	dm-7-RR	RR	YR	dm-7-LR	LR	LY	LW	LF	Total MC
average	47	780	230	41	1700	840	300	580	3400
median	11	85	81	7.6	160	530	100	260	480
maximum	170	4600	1200	230	7900	2300	990	1800	14,000
minimum	4.3	3.8	1.2	0.89	3.0	24	10	8.0	26
*n* detected	8	10	10	10	10	4	4	4	10
*n* not detected	2	0	0	0	0	6	6	6	0

These results confirm that MC-LR, MC-RR and MC-YR are in general more abundant than MC-LY, MC-LW and MC-LF (e.g., [18,20,26]). In our study, MC-LW and/or MC-LF were detected in 25 of the 77 water samples that contained MCs, which means they were present in 32% of the MC-containing samples. This is considerably more than the limited presence of MC-LF and MC-LW found in some studies [20,26], but less than the 45% found in lakes and reservoirs in the United States [18] and the 89% found in a Turkish lake [23]. Also in the American lakes, MC-LY, MC-LW and MC-LF mostly co-occurred, but this was not the case in the Turkish study. 

**Figure 3:**
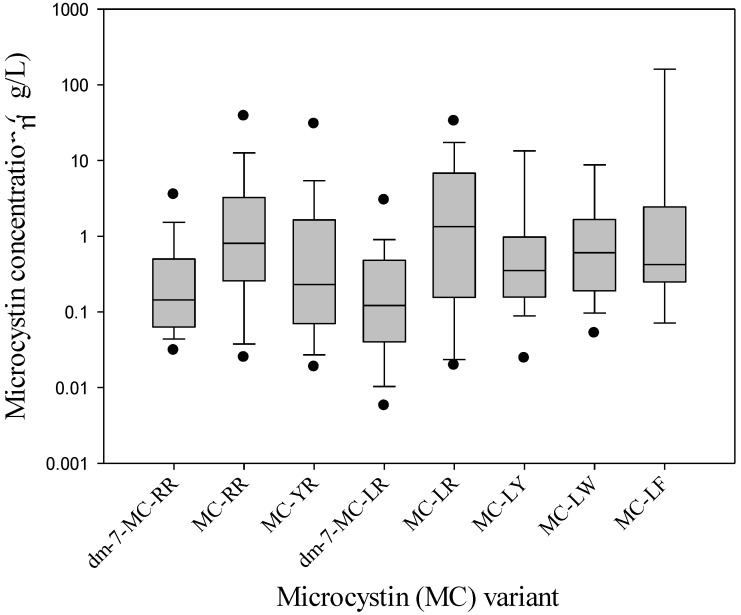
Concentrations of eight different microcystin (MC) variants (μg/L) in 88 water samples from different sites in The Netherlands. Only values above quantification limit are included. Boundaries of the boxes indicate the 25th and 75th percentiles, lines within boxes mark the median, whiskers indicate the 10th and 90th percentiles, dots represent the 5th and 95th percentiles.

The maximum concentration of total MCs in the water samples was 2800 μg/L, and in two scum samples concentrations above 13,000 μg/L were detected (Table 2, Table 3). MC-LR was on average present in the highest concentrations in the water samples, dm-7-MC-RR had on average the lowest concentrations (Table 2, Figure 3). In water samples where MC-LW and MC-LF were present, MC-LW contributed on average 5.2% to the total MC concentration and MC-LF contributed 4.6%. This is slightly higher than the values found in other field samples [17,18]. However, higher relative MC-LW and MC-LF concentrations have been reported from *M. aeruginosa* culture strains, e.g., 21% LW and 19% LF in PCC7820 [32] and 19% LW and 14% LF in AB2005/45 [33]. Those values match with the highest contributions found in our field survey; in the location Borne—t’Dijkhuis, MC-LW contributed for 14% to the total MC-pool, while a 14% contribution of MC-LF was found at Huizen—Gooihoofd (Supplementary Table S1). Thus, the MC variants MC-LW and MC-LF can make up a significant part of the total MC-pool. In one study, extracellular MC-LW was even present at a concentration of 99 μg/L, thereby making up 96% of the total dissolved MC pool in that sample [23]. 

**Figure 4:**
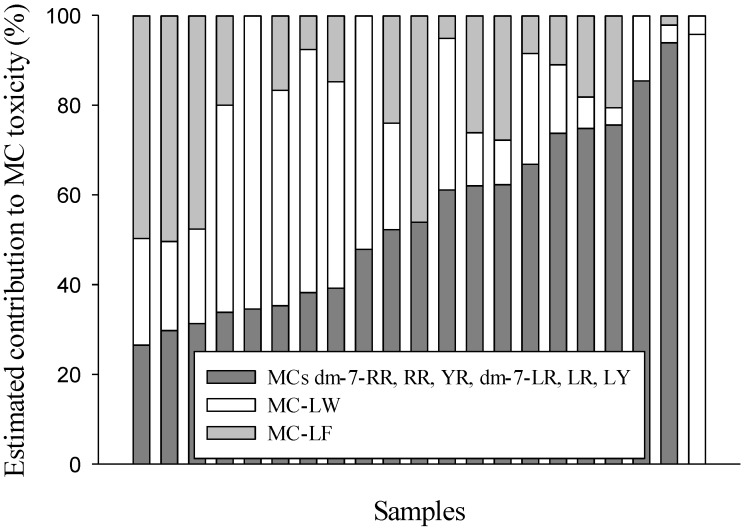
Estimated contribution of MC-LW and MC-LF to the total microcystin (MC) toxicity in 21 surface water samples with cyanobacterial presence.

The contribution of each variant to the overall MC toxicity of a sample was determined by assigning a toxicity factor to each variant (Table 1) and by multiplying variant concentrations by these toxicity factors. Toxicity factors for dm-7-MC-RR, MC-RR, MC-YR, dm-7-MC-LR and MC-LY were estimated from the average i.p. LD_50_ values to mice as given in Zurawell *et al.* (2005) [8]. For MC-LW and MC-LF, the lowest reported *in vitro* toxicity factor to primary human hepatocytes was used [9]. The estimated toxicity contribution of MC-LW and MC-LF in the 21 samples, where these variants were detected above the quantification level, are shown in Figure 4. MC-LW and MC-LF significantly contribute to the overall MC toxicity when they are present; on average 45% of the total MC toxicity is caused by these two variants. It should be noted that these toxicity contributions are estimates and should therefore be interpreted with caution. For six variants, *in vivo* toxicity factors are used, while the contribution of MC-LW and MC-LF was estimated by *in vitro* toxicity factors. It is however unknown whether higher *in vitro* toxicity also results in higher *in vivo* toxicity, as little is known about the toxicokinetics and therefore the bioavailability of the latter variants. It is for instance not known whether MC-LW and MC-LF are stable in the stomach [15]. However, for other variants (MC-RR and MC-LR), comparison of *in vivo* and *in vitro* toxicity data resulted in comparable toxicity factors ([9], Table 1). Also, MC-LW and MC-LF were more toxic than MC-LR to the protozoan *Tetrahymena pyriformis* [16], so it is likely that the enhanced toxicity of MC-LW and MC-LF that was observed *in vitro* will also result in a higher toxicity *in vivo*.

The total toxicity of the samples has only been based on the contribution of the eight MC variants that have been analyzed in this study. As dozens of MC variants have been reported [8] and individual cyanobacterial strains can produce many variants (e.g., [33]), the total toxicity of the samples is likely underestimated. Furthermore, we cannot exclude the possibility that structural isomers, which sometimes exhibit different toxicities [8], have attributed to the signal of some of the eight MC variants analyzed in this study. Nevertheless, our analysis shows that MC-LW and MC-LF are likely to significantly contribute to the total MC toxicity when they are present.

As it has become clear that MC-LW and MC-LF are regularly present in MC containing blooms and that their contribution to the total MC toxicity of these blooms can be substantial, it seems that risk assessment can better be based on the toxicity contributions of all MC variants than only on concentrations of MC-LR, as the first would be a better reflection of the health risk. To assess this risk, concentrations of individual MC variants should be analyzed. However, analytical methods that can identify different variants, such as LC-MS/MS operated in multiple reaction monitoring mode, have the disadvantage of potentially missing variants. Complementary to these methods, total MC concentrations could therefore be determined, for instance by Adda-based ELISA. In this way it can be checked whether the most abundant variants have indeed been identified. For The Netherlands, this would mean that the frequent use of ELISA for determination of MC concentrations should be complemented by analytical methods that can distinguish individual variants.

## 3. Experimental Section

### 3.1. Sample Origin

Samples were collected from 86 fresh surface waters in The Netherlands from 2009 to 2012. All samples were taken in the months July, August, September and October. Locations differed in their morphology from small urban ponds to bigger lakes and sheltered beaches at river banks. Water samples were collected in a polyethylene bottle, scum samples were collected in a small plastic cup. All samples were processed within 24 h of collection.

### 3.2. Sample Extraction

Cyanobacterial species in the samples were identified by light microscopy. Of the water samples, 50 to 250 mL was glass-fiber filtered (GF/C, Whatman International Ltd., Maidstone, UK) and stored at −20 °C. Of each scum sample, 1 mL was filtered over a GF/C filter and also these filters were stored at −20 °C. Before extraction, filters were transferred to 8 mL glass tubes and placed for two hours in a freeze-drier (Alpha 1-2 LD, Martin Christ Gefriertrocknungsanlagen GmbH, Osterode am Harz, Germany). Subsequently, MCs were extracted three times at 60 °C in 2.5 mL 75% methanol–25% Millipore water (v/v). Extracts were dried in a Speedvac (Savant SPD121P, Thermo Scientific, Waltham, MA, USA) and reconstituted in 900 μL methanol. The reconstituted samples were transferred to 2 mL Eppendorf vials with a cellulose-acetate filter (0.2 μm, Grace Davison Discovery Sciences, Deerfield, IL, USA) and centrifuged for 5 min at 16,000× *g* (Galaxy 16DH, VWR, International, Buffalo Grove, IL, USA). Filtrates were transferred to amber glass vials for LC-MS/MS analysis. If needed, samples with high MC concentrations were diluted in methanol before re-analysis.

### 3.3. LC-MS/MS Analysis

Samples were analyzed for eight MC variants (dm-7-MC-RR, MC-RR, MC-YR, dm-7-MC-LR, MC-LR, MC-LY, MC-LW and MC-LF) and nodularin (NOD) by LC-MS/MS as described in [25]. LC-MS/MS analysis was performed on an Agilent 1200 LC and an Agilent 6410A QQQ. The compounds were separated on an Agilent Eclipse XDB-C18 4.6 × 150 mm, 5 μm column by Millipore water with 0.1% formic acid (v/v, eluent A) and acetonitrile with 0.1% formic acid (v/v, eluent B). Elution program was 0–2 min 30% B, 6–12 min 90% B, with a linear increase of B between 2 and 6 min and a 5 min post run at 30% B. Injection volume was 10 μL, flow 0.5 mL/min, column temperature was 40 °C. The LC-MS/MS was operated in positive mode with an electrospray ionisation source, nitrogen was used as drying and collision gas. For each compound, two transitions were monitored in MRM mode. The first quadrupole was operated in unit mode, the second quadrupole was operated in widest mode. Dwell time was 50 ms. MS/MS settings for each compound are shown in Table 4. Calibration standards were obtained from DHI LAB Products (Hørsholm, Denmark) and prepared in methanol, samples were quantified against a calibration curve and subsequently corrected for recovery. Each sample was injected once.

Information on recovery, repeatability, limit of detection and limit of quantification of the analysis is given in [25]. Chromatograms of a calibration standard and a sample are shown in Figure S1.

**Table 4:** MS/MS settings for microcystin (MC) and nodularin (NOD) analysis.

Compound	Retention time	Precursor ion	Fragmentor	Quantifier ion	CE ^1^ quantifier	Qualifier ion	CE ^1^ qualifier	Ratio ^2^
	min	*m*/*z*	V	*m*/*z*	V	*m*/*z*	V	%
dm-7-MC-RR	6.93	512.8	135	135.1	26	70.1	85	1.2
MC-RR	7.62	519.8	151	135.1	30	70.1	75	2.7
NOD	8.03	825.5	220	135.1	70	70.1	95	44.2
MC-YR	8.16	523.3	102	911.5	5	135.1	6	103.6
dm-7-MC-LR	8.21	491.3	88	847.6	5	135.1	6	84.0
MC-LR	8.24	498.3	88	135.1	6	482.3	6	56.7
MC-LY	9.67	868.4	170	163.0	35	136.1	75	29.0
MC-LW	10.22	891.5	146	163.0	31	159.0	75	26.9
MC-LF	10.47	852.5	140	163.1	31	120.1	79	39.1

^1^ Collision energy; ^2^ Ratio between abundance of the qualifier and quantifier ion.

## 4. Conclusions

The MC variants MC-LW and MC-LF were present in 32% of the MC-containing Dutch water samples. When present, these variants contributed on average to nearly 10% of the total MC concentration, but due to their suspected higher toxicity, their average contribution to overall MC toxicity of the samples was estimated to be at least 45%. Given this frequent occurrence and possible high toxicity, it seems better to base health risk estimations on the toxicity contributions of different MC variants than on MC-LR concentrations as is common practice in many countries [30].

## Supplementary Files

Supplementary File 1 Supplementary (PDF, 250 KB)

## References

[1] Carmichael W. (2008). A World Overview—One-Hundred-Twenty-Seven Years of Research on Toxic Cyanobacteria—Where Do We Go from Here?. Cyanobacterial Harmful Algal Blooms: State of the Science and Research Needs.

[2] O’Neil J.M., Davis T.W., Burford M.A., Gobler C.J. (2012). The rise of harmful cyanobacteria blooms: The potential roles of eutrophication and climate change. Harmful Algae.

[3] Codd G.A., Morrison L.F., Metcalf J.S. (2005). Cyanobacterial toxins: Risk management for health protection. Toxicol. Appl. Pharmacol..

[4] Sivonen K., Jones G.J., Chorus I., Bartram J. (1999). Cyanobacterial Toxins. Toxic Cyanobacteria in Water: A Guide to Their Public Health Consequences, Monitoring and Management.

[5] Doekel S., Marahiel M.A. (2001). Biosynthesis of Natural Products on Modular Peptide Synthetases. Metab. Eng..

[6] Dittmann E., Fewer D.P., Neilan B.A. (2013). Cyanobacterial toxins: Biosynthetic routes and evolutionary roots. FEMS Microb. Rev..

[7] Kuiper-Goodman T., Falconer I.R., Fitzgerald J., Chorus I., Bartram J. (1999). Human Health Aspects. Toxic Cyanobacteria in Water: A Guide to Their Public Health Consequences, Monitoring and Management.

[8] Zurawell R.W., Chen H., Burke J.M., Prepas E.E. (2005). Hepatotoxic cyanobacteria: A review of the biological importance of microcystins in freshwater environments. J. Toxicol. Environ. Health Part B.

[9] Fischer A., Hoeger S.J., Stemmer K., Feurstein D.J., Knobeloch D., Nussler A., Dietrich D.R. (2010). The role of organic anion transporting polypeptides (OATPs/SLCOs) in the toxicity of different microcystin congeners *in vitro*: A comparison of primary human hepatocytes and OATP-transfected HEK293 cells. Toxicol. Appl. Pharmacol..

[10] Vesterkvist P.S.M., Misiorek J.O., Spoof L.E.M., Toivola D.M., Meriluoto J.A.O. (2012). Comparative cellular toxicity of hydrophilic and hydrophobic microcystins on Caco-2 cells. Toxins.

[11] Feurstein D., Holst K., Fischer A., Dietrich D.R. (2009). Oatp-associated uptake and toxicity of microcystins in primary murine whole brain cells. Toxicol. Appl. Pharmacol..

[12] Feurstein D., Stemmer K., Kleinteich J., Speicher T., Dietrich D.R. (2011). Microcystin congener- and concentration-dependent induction of murine neuron apoptosis and neurite degeneration. Toxicol. Sci..

[13] Monks N.R., Liu S., Xu Y., Yu H., Bendelow A.S., Moscow J.A. (2007). Potent cytotoxicity of the phosphatase inhibitor microcystin LR and microcystin analogues in OATP1B1- and OATP1B3-expressing HeLa cells. Mol. Cancer Ther..

[14] Feurstein D., Kleinteich J., Heussner A.H., Stemmer K., Dietrich D.R. (2010). Investigation of microcystin congener-dependent uptake into primary murine neurons. Environ. Health Perspect..

[15] Vesterkvist P.S.M., Meriluoto J.A.O. (2003). Interaction between microcystins of different hydrophobicities and lipid monolayers. Toxicon.

[16] Ward C.J., Codd G.A. (1999). Comparative toxicity of four microcystins of different hydrophobicities to the protozoan, *Tetrahymena pyriformis*. J. Appl. Microb..

[17] Cuvin-Aralar M.L., Fastner J., Focken U., Becker K., Aralar E.V. (2002). Microcystins in natural blooms and laboratory cultured *Microcystis aeruginosa* from Laguna de Bay, Philippines. Syst. Appl. Microb..

[18] Graham J.L., Loftin K.A., Meyer M.T., Ziegler A.C. (2010). Cyanotoxin mixtures and taste-and-odor compounds in cyanobacterial blooms from the midwestern united states. Environ. Sci. Technol..

[19] Gambaro A., Barbaro E., Zangrando R., Barbante C. (2012). Simultaneous quantification of microcystins and nodularin in aerosol samples using high-performance liquid chromatography/negative electrospray ionization tandem mass spectrometry. Rapid Commun. Mass Spectrom..

[20] Spoof L., Vesterkvist P., Lindholm T., Meriluoto J. (2003). Screening for cyanobacterial hepatotoxins, microcystins and nodularin in environmental water samples by reversed-phase liquid chromatography-electrospray ionisation mass spectrometry. J. Chromatogr. A.

[21] Ballot A., Krienitz L., Kotut K., Wiegand C., Metcalf J.S., Codd G.A., Pflugmacher S. (2004). Cyanobacteria and cyanobacterial toxins in three alkaline Rift Valley lakes of Kenya—Lakes Bogoria, Nakuru and Elmenteita. J. Plankton Res..

[22] Krienitz L., Ballot A., Kotut K., Wiegand C., Pütz S., Metcalf J.S., Codd G.A., Pflugmacher S. (2003). Contribution of hot spring cyanobacteria to the mysterious deaths of Lesser Flamingos at Lake Bogoria, Kenya. FEMS Microb. Ecol..

[23] Gurbuz F., Metcalf J.S., Karahan A.G., Codd G.A. (2009). Analysis of dissolved microcystins in surface water samples from Kovada Lake, Turkey. Sci. Total Environ..

[24] Craig M., McCready T.L., Luu H.A., Smillie M.A., Dubord P., Holmes C.F.B. (1993). Identification and characterization of hydrophobic microcystins in Canadian freshwater cyanobacteria. Toxicon.

[25] Lürling M., Faassen E.J. (2013). Dog poisonings associated with a *Microcystis aeruginosa* bloom in the Netherlands. Toxins.

[26] Messineo V., Bogialli S., Melchiorre S., Sechi N., Lugliè A., Casiddu P., Mariani M.A., Padedda B.M., Corcia A.D., Mazza R., Carloni E., Bruno M. (2009). Cyanobacterial toxins in Italian freshwaters. Ecol. Manag. Inland Waters.

[27] Wang J., Pang X., Ge F., Ma Z. (2007). An ultra-performance liquid chromatography-tandem mass spectrometry method for determination of microcystins occurrence in surface water in Zhejiang Province, China. Toxicon.

[28] Pavlova V., Babica P., Todorova D., Bratanova Z., Maršálek B. (2006). Contamination of some reservoirs and lakes in Republic of Bulgaria by microcystins. Acta Hydrochim. Hydrobiol..

[29] Lürling M., Faassen E.J. (2012). Controlling toxic cyanobacteria: Effects of dredging and phosphorus-binding clay on cyanobacteria and microcystins. Water Res..

[30] Chorus I. (2012). Current Approaches to Cyanotoxin Risk Assessment, Risk Management and Regulations in Different Countries.

[31] Van De Waal D.B., Verspagen J.M.H., Lürling M., van Donk E., Visser P.M., Huisman J. (2009). The ecological stoichiometry of toxins produced by harmful cyanobacteria: An experimental test of the carbon-nutrient balance hypothesis. Ecol. Lett..

[32] Robillot C., Vinh J., Puiseux-Dao S., Hennion M.C. (2000). Hepatotoxin production kinetics of the cyanobacterium *Microcystis aeruginosa* PCC 7820, as determined by HPLC-mass spectrometry and protein phosphatase bioassay. Environ. Sci. Technol..

[33] Krüger T., Wiegand C., Kun L., Luckas B., Pflugmacher S. (2010). More and more toxins around-analysis of cyanobacterial strains isolated from Lake Chao (Anhui Province, China). Toxicon.

