# Supplementary material for: Occurrence of the Microcystins MC-LW and MC-LF in Dutch Surface Waters and Their Contribution to Total Microcystin Toxicity
Source: Mar Drugs. 2013 Jul 22;11(7):2643–54. doi: 10.3390/md11072643 (PMC3736443; doi:10.3390/md11072643)

Supplementary Information

Table S1. Microcystin concentrations (μg/L) and dominant cyanobacterial species in water samples.

Location	Date	dm-7-RR	RR	YR	dm-7-LR	LR	LY	LW	LF	Total	Dominant cyanobacterial species *
Almere-Witte werf	8/24/2009	0.28	1.1	0.59	0.07	2.3	0.16	-	d.	4.5	M. aer
Almere-Jachthaven	8/31/2009	d.	0.55	0.23	0.14	1.3	-	0.2	d.	2.5	M. aer, Aph. fl-a, An.
Almere-Zwemstrand	8/24/2009	0.36	1.5	0.45	0.25	5.2	0.44	1.1	0.39	9.6	M. fl-a
Almere-Surfschool	8/18/2009	d.	4.9	1.6	0.72	8.9	0.56	1.7	0.55	19	M. aer, Aph. fl-a, An.
Almere-Trailerhelling	8/24/2009	-	0.81	0.13	0.05	1.4	-	-	-	2.3	M. fl-a, M. aer, An. cin, Aph. fl-a
Almere-Zilverstrand	8/3/2009	d.	0.26	0.09	0.03	0.72	-	-	d.	1.1	M. aer, An.
Amersfoort-Vollenhovenkade	8/24/2009	0.27	0.67	35	0.65	19	1.3	0.21	1.2	57	Aph. fl-a, M. wes, M. fl-a, An. fl-a
Andijk-Koopmanspolder	9/15/2009	d.	0.47	0.13	0.04	0.38	-	-	-	1.0	M. aer, Aph. fl-a, An.
Appeltern-Groene Eiland	9/24/2009	-	-	-	-	-	-	-	-	-	none
Appeltern-Gouden Ham	9/24/2009	d.	0.58	0.19	0.03	0.88	-	-	-	1.7	not determined
Asten-Centrum	8/14/2012	-	0.02	d.	-	0.12	-	-	-	0.13	Pico
Beek & Donk-Otterweg	8/14/2012	-	0.03	d.	d.	0.03	-	-	-	0.06	none
Bennekom-Kierkamperweg	8/13/2012	-	-	-	-	-	-	-	-	-	Lim.
Bergen-op-Zoom-Duink. park	8/20/2012	-	-	0.03	d.	0.01	-	-	-	0.05	not determined
Bergen-op-Zoom-Kl. Melanen	8/20/2012	0.06	0.38	0.07	0.01	0.15	-	-	-	0.68	not determined
Bergen-op-Zoom-Vijver Zuid	8/6/2009	-	1.5	0.13	0.19	1.4	-	-	-	3.2	An. fl-a
Borne-t Dijkhuis	7/7/2009	0.59	4.2	0.78	0.62	6.6	-	2.0	-	15	M. aer, M. wes, An. sol, W. nae
Boxtel-Leijensven	8/17/2012	0.09	1.8	0.33	0.08	1.0	-	-	-	3.3	W. nae, An. fl-a, M. wes, M. aer
Boxtel-Essche Heike	8/17/2012	0.03	0.24	0.03	0.00	0.08	-	-	-	0.38	W. nae, M. wes, M. aer
Breda-Linievijver	8/20/2012	-	-	-	-	-	-	-	-	-	none
Budel-Ringelsven	8/14/2012	0.07	0.05	0.02	0.02	0.02	-	-	-	0.18	An., Aph. fl-a
Den Helder-Schoolweg	7/3/2009	0.28	3.3	1.9	0.52	3.7	0.66	-	-	10	Aph. fl-a, M. aer, M. fl-a
Deurne-Burg. Roefslaan	8/14/2012	0.50	12	2.2	0.41	9.1	0.35	0.33	0.24	25	W. nae, M. aer
Dongen-mgr. Schaepmanlaan	8/26/2009	0.63	15	3.4	3.1	24	0.73	1.4	0.49	49	M. aer, W. nae

Table S1. *Cont.*

Ede-Tilanuspark	8/13/2012	-	0.04	d.	d.	0.01	-	-	-	0.05	none
Ede-Jachtlaan	8/13/2012	-	-	-	-	-	-	-	-	-	none
Ede-Kastelenlaan	8/13/2012	0.07	0.07	0.03	0.01	0.03	-	-	-	0.21	Pl. aga
Eijsden-Pieterplas	8/10/2009	d.	1.2	1.2	0.32	6.8	0.26	0.10	0.26	10	M. aer
Eindhoven-Stiffelio	8/14/2012	0.61	0.40	0.08	0.11	0.21	-	-	-	1.4	Pl. aga, M. aer, W. nae
Eindhoven-Stiffelio	8/31/2009	0.14	3.3	0.32	d.	2.1	-	-	-	5.8	M. aer, Aph. fl-a, W. nae, An.
Enschede-Höfteweg	7/7/2009	-	0.26	0.04	0.10	0.08	-	-	-	0.47	An. sol
Etten-Leur-Hoge Neerstraat	8/20/2012	-	-	-	-	d.	-	-	-	d.	An. fl-a
Grave-Anna van Burenweg	8/16/2012	-	-	-	-	-	-	-	-	-	none
Grave-G.W. Loovendaalsingel	8/16/2012	-	-	-	-	d.	-	-	-	d.	Aph. fl-a
Heesch-De Ploeg	8/10/2012	-	-	-	d.	0.02	-	-	-	0.02	none
Heesch-De Ploeg	8/19/2009	1.5	51	3.0	0.81	22	-	-	-	77	M. aer, W. nae
Heesch-Langven	8/10/2012	0.04	0.16	0.03	0.01	0.08	-	-	-	0.31	none
Helmond-Warande	8/14/2012	-	0.03	0.03	d.	0.03	-	-	-	0.09	none
Herten-Oolderplas	7/14/2009	d.	0.14	1.5	0.86	0.99	-	-	-	3.5	M. aer, An.
Huizen-Gooihoofd	7/3/2009	0.35	2.5	5.8	0.92	14	6.0	2.5	5.3	38	M. aer
Huizen-Oostermeent	7/3/2009	1.7	21	41	3.5	71	25	9.5	24	196	M. aer
Huizen-Oostkade	7/3/2009	0.28	3.3	5.8	0.53	12	4.3	1.5	3.5	31	M. aer
Huizen-Westkade	8/24/2009	0.65	5.4	5.3	0.60	19	1.2	1.4	1.4	35	M. aer, An. fl-a
Lelystad-Bovenwater	8/24/2009	-	5.1	4.4	0.21	7.1	-	-	-	17	M. aer
Lelystad-Houtribdijk	8/18/2009	d.	2.1	1.1	0.11	2.51	-	-	d.	5.8	M. aer, Aph. fl-a
Maarheeze-Poelsnep	8/14/2012	-	-	-	-	-	-	-	-	-	none
Medemblik-Recreatieterrein	9/15/2009	d.	0.34	0.11	0.04	0.36	-	-		0.85	M. aer, Aph. fl-a, An.
Middelrode-Christinastraat	8/10/2012	0.05	0.56	0.03	0.47	16	0.51	0.10	0.05	17	An. pla, Aph. fl-a, M. aer
Naarden-Meertje van Vlek	8/24/2009	0.05	0.02	0.04	0.22	9.2	0.001	-	-	9.5	M. fl-a
Nijkerk-Nieuw Hulckestein	9/14/2009	d.	0.07	0.04	0.04	0.27	-	-	-	0.42	M. aer, Pl. aga, Aph. fl-a, An.
Nijmegen-Bizonbaai	9/24/2009	d.	0.76	0.23	0.74	1.5	0.08	-	d.	3.3	M. aer, W. nae, Aph. fl-a

Table S1. *Cont.*

Ohé en Laak-Dilkensplas	8/10/2009	-	d.	d.	-	0.03	-	-	-	0.03	M., An., Aph., W. nae, C.
Oldenzaal-Griekenlandweg	7/7/2009	3.6	66	24	2.5	56	-	-	-	152	M. aer, An. fl-a
Oldenzaal-Harplaan	7/7/2009	-		0.07	0.05	0.02	-	-	-	0.15	not determined
Oldenzaal-Thijsniederweg	7/7/2009	0.94	18	2.8	0.40	7.7	-	-	-	30	M. aer, Pseu., Aph. fl-a
Onderdijk-Drogewijmers	9/1/2009	-	-	-	-	-	-	-	-	-	An.
Onderdijk-Vooroever	9/15/2009	d.	0.51	0.14	0.05	0.48	-	-	-	1.2	M. aer, Aph. fl-a, An.
Ooltgensplaat-Dagstrand	9/9/2009	d.	0.18	0.09	0.01	0.16	-	-	-	0.44	M. aer
Oost-Maarland-Dagstrand	8/10/2009	d.	0.90	0.72	0.25	6.4	0.48	0.19	0.42	9.3	M. aer
Plasmolen-Mookerplas	9/28/2009	0.14	3.2	1.7	0.95	6.9	0.21	0.05		13	M. aer
Rilland-Kreekraksluizen	9/14/2009	d.	d.	0.02	-	0.02	-	-	-	0.04	M. aer
Roosendaal-Dadelberg	8/20/2012	-	-	-	-	-	-	-	-	-	not determined
Roosendaal-Dubbelberg	8/20/2012	-	-	-	-	-	-	-	-	-	not determined
Roosendaal-Enclaveberg	8/20/2012	0.36	9.9	2.2	0.16	6.0	-	-	-	19	M. aer, M. wes, W. nae
Schijndel-Ren. Rubinsteinlaan	8/10/2012	-	-	-	d.	-	-	-	-	d.	not determined
St-Michielsgestel-Goudplevier	8/10/2012	-	-	-	-	-	-	-	-	-	none
Sint-Oedenrode-Kienehoef	8/5/2009	-	0.48	0.11	0.12	0.25	-	-	-	0.96	not determined
Sint-Oedenrode-Molenwiel	7/27/2012	0.47	2.8	0.33	0.12	2.1	0.12	0.54	0.08	6.6	Aph. fl-a, W. nae, M. aer
Sint-Oedenrode-Molenwiel	8/17/2012	0.12	0.73	0.37	0.04	0.55	-	-	-	1.8	Aph. fl-a, M. aer
Someren-Van Gijselstraat	8/14/2012	0.12	0.85	0.08	0.03	1.1	0.10	-	0.16	2.5	W. nae, An. fl-a
Son-Europalaan	8/14/2012	0.05	0.11	0.01	0.003	0.03	-	-	-	0.21	Aph. fl-a, Pl. aga, An. pla
Spakenburg-t Kleine Zeetje	8/24/2009	0.11	0.28	0.19	0.01	0.73	-	-	-	1.3	M. aer, Aph. fl-a, An.
Speelmansplaten-Oesterdam	9/21/2009	d.	1.3	1.1	0.07	1.5	-	-	-	4.0	M. aer
Stichtse brug-Gooimeerzijde	7/6/2009	d.	0.61	1.6	0.10	3.7	0.30	0.10	0.28	6.7	M. aer, An.
Tilburg-Bergland	8/17/2012	0.06	0.42	0.04	0.03	0.75	-	-	-	1.3	W. nae, M. wes, M. aer
Tilburg-Kaukasus	8/17/2012	0.27	5.8	0.48	0.23	4.3	0.26	-	-	11	M. wes, M. aer, W. nae
Tilburg-Stappegoor	8/17/2012	-	0.03	0.02	d.	0.02	-	-	-	0.06	Pico
Tilburg-Reggevijver	8/17/2012	0.13	1.9	0.21	0.36	5.0	0.09	-	-	7.7	An. fl-a, M. aer, W. nae

Table S1. *Cont.*

Tilburg-Essche Stroomvijver	8/17/2012	0.05	0.13	0.03	0.01	0.25	-	-	-	0.47	W. nae
Tilburg-Hoge Witsie	8/17/2012	-	-	d.	d.	-	-	-	-	d.	Pl. aga
Tilburg-Quirijnstokpark	8/6/2009	-	-	-	-	0.05	-	-	-	0.05	none
Urk-Westhavendam	8/4/2009	d.	1.3	0.33	0.14	3.1	0.15	0.67	0.29	6.0	M. aer, Aph. fl-a
Valkenswaard-Dragonder	8/14/2012	0.04	0.02	0.02	0.04	0.24	-	-	-	0.37	An., Aph. fl-a
Wageningen-Dreyenvijver	8/13/2012	0.06	0.92	0.08	0.01	0.18	-	-	-	1.3	W. nae, M. aer
Wageningen-Lumen	8/20/2009	-	-	-	-	-	-	-	-	-	An. spi, An. fl-a
Wessem-Koeweide	8/25/2009	d.	4.4	1.7	0.11	6.8	0.19	0.19	d.	13	M. aer, An.
Wierden-Amstelmeer	10/2/2011	2.1	63	4.5	220	2100	110	260	33	2800	M. aer
Zeewolde-Laakse strand	8/31/2009	d.	5.7	1.8	0.43	7.1	-	-	-	15	M. aer, Aph. fl-a, An.
	average	0.45	5.0	2.4	4.0	35	5.9	14	4.2	49	
	median	0.20	0.81	0.23	0.12	1.3	0.35	0.61	0.42	2.5	
	maximum	3.6	66	41	220	2100	110	260	33	2800	
	minimum	0.03	0.02	0.01	0.003	0.01	0.001	0.05	0.05	0.02	
	n quantified	38	67	67	62	73	25	20	17	73	
	n not quantified	20	2	5	9	2	0	0	6	4	
	n not detected	30	19	16	17	13	63	68	65	11	
	average	0.45	5.0	2.4	4.0	35			4.2	49	

***** An.: *Anabaena*, An. cir: *A. circinalis*, An. fl-a: *A. flos-aquae*, An. pla: *A. planktonica*, An. sol: *A. solitaria*, Aph.: *Aphanizomenon*, Aph. fl-a: *A. flos-aquae*, C.: *Cylindrospermopsis*, Lim: *Limnothrix*, M.: *Microcystis*, M. aer: *M. aeruginosa*, M. fl-a: *M. flos-aquae*, M. wes: *M. wesenbergii*, Pico: picocyanobacteria, Pl. aga: *Planktothrix agardhii*, Pseu: *Pseudoanabaena*, W. nae: *Woronichinia naegeliana*.

Table S2. Microcystin concentrations (µg/L) and dominant cyanobacterial species in scum samples.

Location	Date	dm-7-RR	RR	YR	dm-7-LR	LR	LY	LW	LF	Total	Dominant cyanobacterial species*
Budel-Ringelsven	8/14/2012	4.3	14	2.4	0.89	11	-	-	-	32	An. fl-a, An. pla
Deurne-Burg. Roefslaan	8/14/2012	130	4600	290	230	6000	1000	990	510	14,000	W. nae, M. aer
Ede-Kastelenlaan	8/14/2012	5.6	8.6	6.4	2.7	3.0	-	-	-	26	Pl. aga
Eindhoven-Stiffelio	8/31/2009	170	2500	400	65	2100	24	73	-	5300	M. aer, An. fl-a, Aph. fl-a, An. sol, M. wes
Sint-Oedenrode-Molenwiel	8/24/2012	41	280	150	13	240	38	10	10	790	Aph. fl-a, M. aer, An. fl-a
Sint-Oedenrode-Molenwiel	8/17/2012	15	250	240	6.8	190	-	-	-	700	Aph. fl-a, M. aer
Someren-van Gijsselstraat	8/14/2012	5.2	57	3.6	1.2	55	-	-	8.0	130	W. nae, An. fl-a
Tilburg-Reggevijver	8/17/2012	7.3	110	8.4	8.5	120	-	-	-	260	W. nae, M. aer, An. fl-a
Valkenswaard-Dragonder	8/14/2012	-	3.8	1.2	3.8	25	-	-	-	34	An. cir, Aph. fl-a
Wageningen-Onderlangs	9/17/2012	-	7.0	1200	79	7900	2300	130	1800	13,000	M. aer
	average	47	780	230	41	1700	840	300	580	3400	
	median	11	85	81	7.6	160	530	100	260	480	
	maximum	170	4600	1200	230	7900	2300	990	1800	14,000	
	minimum	4.3	3.8	1.2	0.89	3.0	24	10	8.0	26	
	n detected	8	10	10	10	10	4	4	4	10	
	n not detected	2	0	0	0	0	6	6	6	0	

Figure S1. LC-MS/MS chromatograms of calibration standards (**A**) and a ten times diluted water sample from location Wierden-Amstelmeer (**B**). Transitions for the same compounds are shown in the same colour, transition for the quantifier ion are represented by a bold line, transitions for the qualifier ions are represented by a normal line.

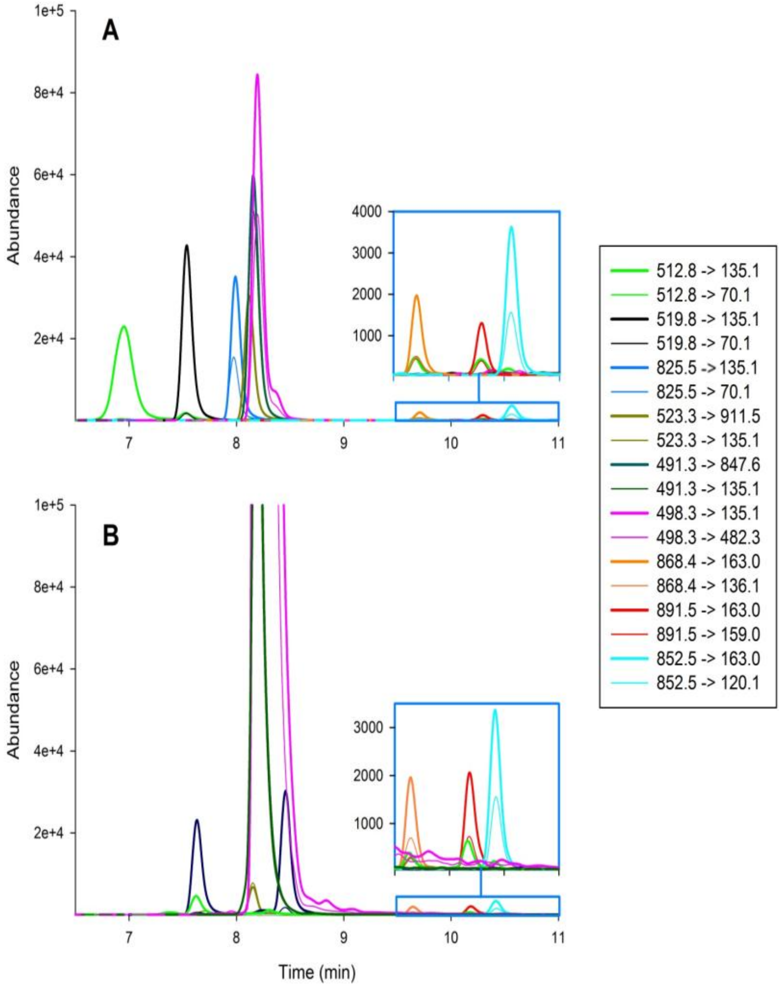

Supplement: Supplementary File 1 — Supplementary (PDF, 250 KB) [file marinedrugs-11-02643-s001.pdf]
